# Ontario Veterinary College—Commencement

**Published:** 1892-04

**Authors:** 


### ONTARIO VETERINARY COLLEGE—COMMENCEMENT.

The Annual Commencement exercises of the Ontario Veterinary College were held in the lecture theatre of the college on Temperance Street. The chair was taken by Dr. Andrew Smith, Principal of the institution, among those supporting him being Hon. John Dryden, Minister of Agriculture; N. Awrey, M. P. P , President of the Agriculture and Arts Association; Dr. Daniel Clark; Hon. Thomas Ballantyne, Speaker of the Legislative Assembly; Rev. D. J. Macdonnell, Dr. Thorburn, Dr. McMahon, M. P. P., Wm. Christie, Rev. Dr. Briggs: Henry Wade, Secretary Agriculture and Arts Association; Inspector J. L. Hughes, Ald. Score, Mayor Lloyd, Newmarket, and others.

In opening the proceedings, Dr. Smith stated that this was the 22nd anniversary of the institution, and the closing session had been one of the most successful in its history, during which students to the number of 350 had been present from all parts of Canada and the States and even from the United Kingdom. The graduates were: Richard Henry Alexander, Strathroy; James L. Armitage, Lucan; James M. Armstrong, Locust Hill. George Beacon, Clinton; Wm. J. Badgley, Collamer, N. Y.; Wm. H. Barry, Omagh; Howard L. Baum, Shelly, Pa.; Wm. G. Birdsall. Peterboro'; Charles N. Blanshard, Appleby; J. H. L. Blattenburg. Smithville, Ohio; Samuel D. Bodle, Mecklenburg, N Y.; Frra La., Botkin, Union Port. Ind.; Alex. G. Bowker, Norfolk, Eng.; Robert L. Bradley, Louisville, Ky.; Fred. Bragington, Crown Point, Ind.; T. H. Buckingham, Elkhorn, Man.; Philip G. Button, Cresco, Iowa; L. C. Brewster, Belleville; Asa A. Brown, Toronto: Fred. C. Brown, Rodney; Hosea B. Crandall, Syracuse, N. Y.; Alva B. Carter, Brocton, Ill.; William S. Cass, Raymond, Ill.; Edward M. Clarke, Bishop, Cal.; Nathaniel Clark, Mount Brydges; Richard Coghlan, Strathroy; Robert Colgan, St. Catharines; Horace H.

Collins, Obold, Pa.; Samuel A. Coxe, Brandon, Man.; A. H. Crane, London; Samuel R. Craver, Warren, Ohio; David W. Curtis, Listowel; Ashton B. Cutcliffe, Mohawk; Harry R. Church, Luzerne, Pa.; Charles H. Daniels, North Adams, Mass.; L. Enos Day, Concordia, Kansas; Gravier G. Dean, Tully, N. Y.; David C. Detwiler, Ironbridge, Pa.; James S. Elliott, Gosport, N. Y.; John J. Elliott, Wingham; C. H. Erganbright, North Salem, Ind; Peter M. Fisher; Georgetown; E. D. Fisher, Skaneateles, N. Y.; Ernest Foreman, Jr., Whitehall, Ill.; R. H. Fortune, Vesta; Harry Fulstow, Greenwich, Ohio; Leslie A. Gemmel, Islington; Andrew D. Gemmill, Wingham; Samuel Goldsmith, Reading, Pa.; James H. Gregory, Danville, Pa.; John W. Griffith, Pitpon, Iowa; George E. Grover, Chatham; John S. Grove; Nimisila, Ohio; Adolphus W. Guest, London, Peter F. Gaunt, Kinkora; Alfred T. Goldie, Corunna; William J. Hallock, Danby, N. Y.; William F. Harding, New Milford, Pa.; Leonard G. W. Hart, Eau Claire, Wis.; Henry J. Hellwig, Elliston, Ohio; James H. Hester, Hastings, Nebraska; Harry E. Hurd, Brockville; Benjamin F. Huston, Cortland, Neb.; Henry H. Hawley, Niagara Falls, N. Y.; Wilfred T. Hart, Ravena, Ohio; Frank A. Ingram, Springfield, Mass.; Thomas W. Johnston, L'Amaroux; T. E. Jago, Rockwood; Frank C. Jervis, Cambridgeboro, Pa.; John L. Kolander, Pewaukee, Wis.; William Kinney, Cedar Valley, Ohio; George Kesler, McKeesport, Pa.; E. G. Kriebel, Hereford, Pa.; C. W. Kuhn, Mercersburgh, Pa.; Thomas J. Lawson, Logoch; William J. Lawson, Cherrywood; Louis D. LeGear, Inlay City, Mich.; Micheal Little, Pilot Mound, Man.; John H. Lipsett, Brandon, Man.; Kenneth J. McKenzie, Northfield, Minn.; Thomas McFarlane, Ottawa; Frank H. McCarthy, Pottsville, Pa.; Thomas W. McConnell, Moline, Mich.; Daniel McCuaig, Bristol, Que.; T. F. McEvers, Chicago, Ill.; Ashton D. McKenney, Waterdown, S. Dak.; J. H. McLevey, Florence; Wallace McQueen, Simcoe; John E. Miller, Greenville, Texas.; George W. Milligan, Fairbault, Minn.; William Mock, Easton, Pa.; Harvey Campbell Moore, Deer Park; John O. Moore, Durham; Robert J. Morrison, Detroit, Mich.; Charles M. Noble, Monticello, Iowa; E. H. Nodyne, Rochester, N. Y.; John H. O'Brien (M. D.), Rochester, Pa.; George W. Orchard, Strathroy; Williamm H. Orth, Wallaceburg; David C. Papworth, Syracuse, N. Y.; Walter J. Payne, West Walworth, N. Y.; Wm. O. Penney, Louisville, Ky.; N. B. Perdue, Orangeville; James Pickel, St. Mary's; J. W. Poole, Crystal City, Man.; Robert Rives, Greenfield, Ill.; Thomas E. Robinson, Glastonbury,

Conn.; Henry Roome, Croydon, Eng.; Joseph Routledge, Lucan; Henry A. Rudd, Dublin, Ireland; Ellerton A. Richardson, Strathroy; Joseph S. Schofield, Naugatuk, Conn.; James L. Savage, Chesley; Joseph P. Sherman, Detroit, Mich.; Wm. A. Shoults, Portage la Prairie, Man.; Wm. H. Simmons, London, Eng.; Fulcard C. Smock, Carltonville, Ill.; Samuel Sommerville, Buffalo, N. Y.; Warren E. Stocking, Eagle Harbor, N. Y.; Frank W. Swearingen, Decatur, Ill.; James L. Smith, Strathroy; Thomas Trinder, Cork, Ireland; Richard W. Tuck, Elgin, Ill.; Joseph W. Turner, Uxbridge; John S. Thompson, Darlington, Wis.; J. N. Umphrey, Udora; James A. Wake, Moosomin, Assa.; Walter Warren, Windsor, Mo ; J. Will Watson, Peru, Ind.; Robert H. Watson, Chicago, Ill.; Benjamin F. Wingard, Bryan, Ohio; Thomas C. Wallace, Jamestown, N. Y.; Ben. P. Wende, Millgrove, N. Y.; Thomas C. Young, Bristol, Que.; William G. Zimmerman, Smithville, Ohio.

The rewards for best general examination—F. L. Botkin, Union Port, Ind., U. S.; gold medal presented by the Ontario Veterinary Association. Honors—G. Beacon, J. H. Hester, E. A Richardson, W. A. Shoults, F. W. Swearingen.

Special prize—Presented by W. Christie, trustee of Toronto University, to student taking the greatest number of prizes—F. W. Swearingen, complete set of Darwin's works.

The Board of Examiners consisted of the following gentlemen —J. H. Wilson, V. S., London; W. Cowan, V. S., Galt; J D. O'Neill, V. S., London; C. Elliott, V. S., St. Catharines; T. H. Lloyd, V. S., Newmarket; W. Shaw, V. S., Dayton, Ohio, U. S.; C. H. Sweetapple, V. S., Toronto; J. Thorburn, M. D., Toronto.

Addresses were read by Hon. Thos. Ballantyne, Hon. John Dreyden, Minister of Agriculture, and the president of the Agriculture and Arts Association, Mr. Nicholas Awrey, M. PP.

Dr. Daniel Clark, medical superintendent of the Toronto Asylum for the Insane, urged the men to give special attention to the study of mental diseases in animals. Animals had minds and brains, and gave constant and abundant evidence of reasoning powers of a very high order. He believed that often when an animal was called vicious or bad-tempered its conduct was due to an affectation of the brain; a very notable instance of this was that of the big elephant Jumbo, whose violeut fits of unreasoning temper tallied exactly with the symptoms observed in human beings who were mentally afflicted. This was practically an unexplored territory, a terra incognita, and he urged some of the students to take up the study.

